# Inducing Dzyaloshinskii–Moriya interaction in symmetrical multilayers using post annealing

**DOI:** 10.1038/s41598-022-16244-w

**Published:** 2022-07-13

**Authors:** Khadijeh Ahmadi, Farzad Mahfouzi, Loghman Jamilpanah, Morteza Mohseni, Tobias Böttcher, Philipp Pirro, Nicholas Kioussis, Johan Åkerman, S. A. Seyyed Ebrahimi, Seyed Majid Mohseni

**Affiliations:** 1 Department of Physics, Shahid Beheshti University, Evin, Tehran, 19839 Iran; 2 Advanced Magnetic Materials Research Center, School of Metallurgy and Materials, College of Engineering, University of Tehran, Tehran, 11155 4563 Iran; 3 Department of Physics and Astronomy, California State University Northridge, Northridge, CA 91330-8268 USA; 4 Fachbereich Physik and Landesforschungszentrum OPTIMAS, Technische Universit¨at Kaiserslautern, 67663 Kaiserslautern, Germany; 5 Materials Physics, School of Information and Communication Technology, KTH Royal Institute of Technology, Electrum 229, 164 40 Kista, Sweden; 6 Department of Physics, University of Gothenburg, 412 96 Gothenburg, Sweden

**Keywords:** Nanoscience and technology, Physics

## Abstract

The interfacial Dzyaloshinskii-Moriya Interaction (iDMI) is an antisymmetric exchange interaction that is induced by the broken inversion symmetry at the interface of, e.g., a ferromagnet/heavy metal. Thus, the presence of iDMI is not expected in symmetrical multilayer stacks of such structures. Here, we use thermal annealing to induce the iDMI in a [Py/Pt]_×10_ symmetrical multilayer stack. Brillouin light scattering spectroscopy is used to directly evidence the iDMI induction in the annealed sample. Structural characterizations highlight the modified crystallinity as well as a higher surface roughness of the sample after annealing. First principles electronic structure calculations demonstrate a monotonic increase of the iDMI with the interfacial disorder due to the interdiffusion of atoms, depicting the possible origin of the induced iDMI. The presented method can be used to tune the iDMI strength in symmetric multilayers, which are the integral part of racetrack memories, magnonic devices as well as spin-orbitronic elements.

## Introduction

The Dzyaloshinskii-Moriya Interaction (DMI) is an antisymmetric exchange interaction that appears in inversion asymmetric structures and favors perpendicular alignment of neighboring spins in a magnetic material^1^. The DMI can lead to the formation of stable chiral spin textures such as Néel-type domain walls or skyrmions^2,3^, which are promising candidates for memory and logic applications^4,5^. In ferromagnet/heavy metal (FM/HM) heterostructures, the large spin–orbit coupling (SOC) of the HM and the broken inversion symmetry at the interface result in an interfacial Dzyaloshinskii–Moriya interaction (iDMI)^6^.

For real applications e.g. racetrack memory devices, the DMI is demanded in multilayers or superlattices with both symmetric and asymmetric structures^7,8^. However, the DMI vanishes in the symmetric structures due to the symmetry of the stacks, therefore, finding ways to induce the DMI in such structures is highly desired.

So far, several methods have been used for this purpose. For example, it has been reported that the iDMI is realized in such magnetic superlattices due to the gradience in the interface quality which is induced during the growth of the layers^2,3^. Moreover, it has been shown that a finite iDMI can be observed if the upper and lower interfaces are of different crystallographic quality in a symmetric Pt/Co/Pt stack^9–11^. This highlights the importance of the structure and the relative interface morphology of the upper and lower FM interfaces in determining the iDMI in such systems^12^.

In this context, several studies have focused on the induction or tuning of the DMI in thin film structures via surface engineering. For example, Zimmermann et al*.*^13^ used density-functional theory calculations to show the possibility to modify the magnetic properties at Co/Pt bilayer interfaces with chemical disorder based on the intermixing of atoms at the interface. Furthermore, the influence of the interface quality, considering the surface roughness and atomic intermixing on the iDMI has been studied in symmetric Pt/Co/Pt structure by Wells et al*.*^12^. They found that the net iDMI increases as the difference between the interface quality of the top and bottom Co layer increases. In addition, Samardak et al.^14^ studied the effect of atomic-scale surface modulation on the iDMI in ultrathin films composed of 5d heavy metal/ferromagnet/4d (5d) heavy metal or oxide interface. They predicted that a significant enhancement of the iDMI by up to 2.5 times can be achieved through interface roughness engineering on the atomic scale. Moreover, Torrejon et al.^15^ have found that the strength of the iDMI varies by changing the 5d orbitals filling, in other words, the electronegativity of the heavy-metal layer that is used in the structure.

In this article, we use thermal annealing to induce the iDMI in a [HM/FM/HM]_10_ symmetrical multilayer stack. Structural characterizations show that the thermal annealing results in the interfacial microstructure evolution and roughness which causes the induction of the iDMI. Direct observation of the iDMI is performed by Brillouin light scattering (BLS) spectroscopy measurement. To support the measurements, we carried out first-principles calculations which show a monotonic variation of the iDMI with the interfacial disorder due to interdiffusion of atoms. In order to study effects of the interface, we suggested substitutional disorder at the interface by switching the positions of Ni and Pt atoms and also the concentration of Fe atoms at the interface. Our method, as a post treatment to the grown samples gives the opportunity of large qualitative control of the treatment without intensive instrumentation. This method is also cheap and can motivate research on thermal treatment in different ambient by controlling qualities like gas type and pressure which might give result in new pathway for optimization of spintronic and magnonic devices.

## Experimental results and discussion

**Figure 1:**
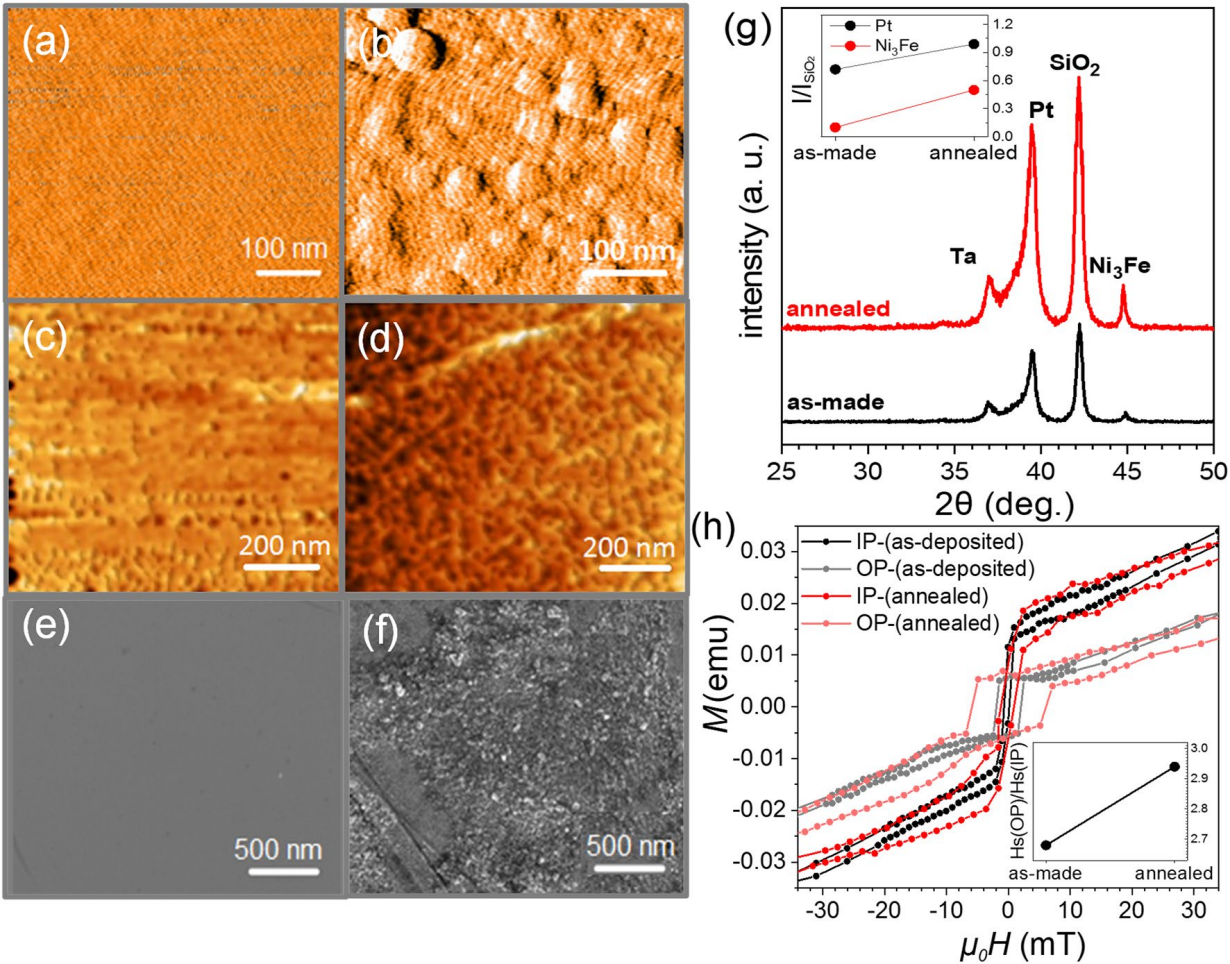
(**a**–**b**) The AFM images, (**c**–**d**) MFM images and (**e**–**f**) SEM images of the as-deposited and annealed samples respectively. (**g**–**h**) The XRD pattern and, the in-plane and out of plane hysteresis loops before and after annealing.

A multilayer stack with Ta(5 nm)/Pt(6 nm)/[Ni_80_Fe_20_(3 nm)/Pt(1 nm)]_×10_/Au(3 nm) structure was deposited on a thermally oxidized Si substrate at room temperature by DC magnetron sputtering. We first investigate the structural characteristics of the as-deposited and the annealed samples to understand the impact of the annealing on the microstructure of the stacks. Figure 1a,b show the atomic force microscopy (AFM) images of the as-deposited and annealed samples, respectively. The surface roughness is about 0.5 nm for the as-deposited while it is increased to approximately 5 nm for the annealed sample. The magnetic domain patterns which are measured by the magnetic force microscopy (MFM) for the as-deposited and annealed samples without an external field, are presented in Fig. 1c,d, respectively. In the as-deposited sample, as demonstrated by the uniform color contrast, the magnetization is in the plane, while in the annealed sample, the magnetic domains formed in a labyrinthine pattern which can indicate that domains do not orientate purely in the plane^16^. Field emission scanning electron microscopy (FESEM) was employed to characterize the structural and morphological properties. In Fig. 1e,f which display the FESEM images, one can observe that the as-deposited sample is uniform, however after annealing, the film surface is rough and planar nano-sized grains are formed. In general, from surface morphology characterizations, we conclude that a higher surface roughness can be observed for the annealed sample which can affect the iDMI consequently.

The X-ray pattern of the samples, presented in Fig. 1g indicate that Ni_80_Fe_20_ (Py) forms a face-centered-cubic structure of the type Ni_3_Fe (Reference code: 96–90-3942, Space Group: Pm-3 m, 221) in both as-deposited and annealed samples. The intensity of Py and Pt peaks are more pronounced in the annealed samples. This indicates that the degree of crystallinity for the Py and Pt layers are appreciably improved after annealing. The improved crystallinity of the annealed sample is important for the presence of iDMI at the interfaces, which will be discussed later.

The hysteresis loops of the samples which are measured for the in-plane (IP) and out-of-plane (OP) configurations using a Vibrating-sample magnetometer (VSM) system are presented in Fig. 1h. It can be seen that neither the as-deposited nor the annealed samples have a purely in-plane or an out-of-plane magnetic anisotropy, that highlights the reorientation of the magnetization. Moreover, the coercivity of the sample is increased after annealing which can be due to the higher crystallinity of this sample as well as the interface sharpening^17,18^. In these multilayers, the sharp interfaces between the magnetic and non-magnetic lattices leads to the hybridization of the magnetic metal’s 3d and non-magnetic metal’s 5d orbitals, which can tune the direction of the magnetic anisotropy depending on the orbital bindings^19–22^. In our sample, this anisotropy axis is more tilted towards the film plane after annealing, which is evidenced by the OP/IP relative saturation fields (*H*_s_) observed from the VSM data. This relative increase is depicted at the inset of Fig. 1h. Briefly, it is the easy axis, which rotates towards the film plane.

We now turn our focus to magnetic properties of the samples. First, the spin-waves (SWs) spectroscopy using BLS is used to measure the iDMI strength in both samples. An in-plane bias magnetic field sufficient to saturate the sample is applied to allow the SWs to propagate in the film plane. The iDMI leads to a frequency non-reciprocity of counter-propagating magnetostatic surface SWs (aka Damon-Eshbach mode). Such a frequency non-reciprocity can be observed by the difference between the Stokes (S) and anti-Stokes (AS) frequencies in the BLS spectroscopy^23,24^. The same argument applies once the direction of the applied field is inverted. The measured frequency non-reciprocity with respect to the wave vector can be easily characterized, which depends on the DMI constant (*D)* based on the $\Delta$ *f* = $[2\gamma /{\pi M}_{s}$] *D k*_sw_, where $\gamma$ is the gyromagnetic ratio and *M*_*S*_ is the saturation magnetization^24^. It is required to probe surface waves, for which iDMI-induced nonreciprocity becomes maximal and, in particular, linear in k with DMI constant**.**

**Figure 2:**
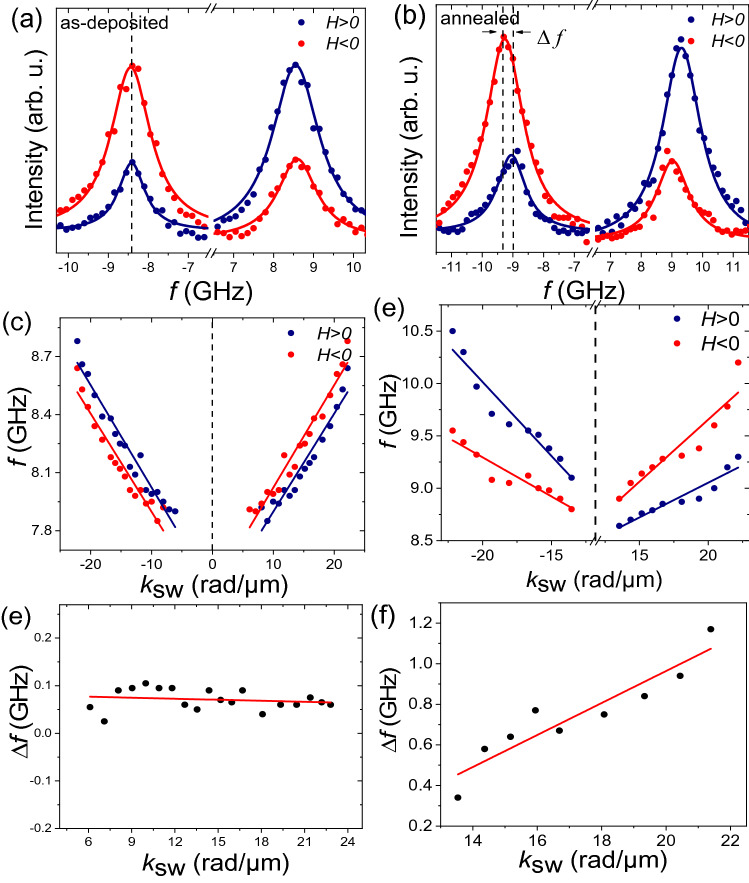
Results from the Brillouin light scattering spectroscopy. (**a**–**b**) The measured BLS spectra of the SWs having wave number of *k*_*sw*_ = 20.44 rad/*μ*m in the presence of two different applied field values (${\mu }_{0}$ *H* =  ± 50 mT) for the as-deposited and annealed samples, respectively. Symbols refer to the experimental data and solid lines are the Lorentzian fits. (**c**–**d**) The measured dispersion relation of the SWs including the Stokes (S) and anti-Stokes (AS) peaks for the as-deposited and annealed samples respectively. Symbols and solid lines correspond to the experimental data and linear fits results. (**e**–**f**) The frequency difference $\Delta$ f between the counter-propagating SWs as a function of the wave vector for the as-deposited and annealed samples, respectively.

The BLS spectra of the SWs having wavenumber of *k*_*sw*_ = 20.44 rad/μm in the presence of an applied field of ${\mu }_{0}$ *H* =  ± 50 mT (with opposite direction) are shown for the as-deposited and annealed samples in Fig. 2a,b respectively. The BLS spectrum of both field directions shows a SW mode. The annealed sample shows a different SW characteristic. The results from the Lorentzian fits show that the frequency non-reciprocity for this wavenumber is as large as Δ*f* = 490 MHz.

In order to understand the nature of the observed SW mode, we present the measured SW dispersion relation. Figure 2c,d shows frequency of the S and AS peaks as a function of the wave vector *k*_sw_, at ${\mu }_{0}$ *H* =  ± 50 mT for the AS and S modes for the as-deposited and annealed samples respectively. Indeed, the SW dispersion relation is reciprocal, i.e. the AS and S peaks for a given wavenumber have similar frequency for the as-deposited sample. From Fig. 2d, there is frequency difference between counter-propagating SWs for both orientations of the applied magnetic field for the annealed sample.

Figure 2e shows the frequency non-reciprocity (∆*f*) with respect to the wave vector for the as-deposited sample. A vanishing of the frequency difference is observed, indicating that the iDMI is absent in this sample. The sign and the amplitude of the iDMI constant *D*, have been obtained from the measured frequency non-reciprocity which is presented in Fig. 2f for the annealed sample^23,25^. The results show a linear variation of the frequency non-reciprocity (∆*f*) with respect to the wave vector. The slopes obtained from a linear fit of this variation is 0.07881 $\pm$ 0.011 GHz/radµm^-1^. From the slope of the curve, we estimate the value of the *D* by assuming $\gamma$ =185 rad/T^-1^ ns^-1^^26,27^ and *M*_S_ = 700 kA/m for Py in our samples^28^. Therefore, the estimated value of the iDMI constant from BLS measurements is *D* = 0.47 $\pm$ 0.02 mJ*/*m^2^. The obtained iDMI constant is positive (*D* $>$ 0), thus, it favors a right-handed chirality^29^.

As the structural characterizations show the crystal related evolution by annealing and also the BLS results show the evolution in iDMI, therefore, for a better evaluation on the possible origin of the induced iDMI, computational study were performed for different interface conditions as the following.

## Computational results

The iDMI is particularly sensitive to the structural changes of the layers, especially at the interfaces. This includes processes such as crystallization or interdiffusion at the interfaces. Therefore, the improved ordering of the atoms at the Pt/Py interface, due to the annealing treatment, can be the reason for the modification of the iDMI^9^^,^^30^. In order to support this hypothesis, we have carried out first-principle calculations, discussed in detail below.

The first principles calculations of DMI coefficients are carried out using relativistic exchange couplings^31^ calculated from the Hamiltonian determined from density functional theory calculations employing the OpenMX^32,33^ ab initio package. We adopted Troullier-Martins type norm-conserving pseudopotentials^34^ with partial core correction. We used a 24 $\times$ 24 $\times$ 1 k-point mesh for the first Brillouin zone (BZ) integration, and an energy cutoff of 500 Ry for numerical integrations in the real space grid. The localized orbitals were generated with radial cutoffs of 6.0 a.u, 6.0 a.u and 7.0 a.u. for Ni, Fe and Pt, respectively^32^^,^^27^.

Structural relaxations were carried using the Vienna ab initio simulation package (VASP)^35,36^ within the generalized gradient approximation as parameterized by Perdew et al.^37^. The pseudopotential and wave functions are treated within the projector-augmented wave method^38^. A 5 Å thick vacuum region is introduced to separate the periodic slabs along the stacking direction. The plane wave cutoff energy was set to 500 eV and a 6 $\times$ 6 $\times$ 1 k-points mesh was used in the 2D Brillouin zone sampling.

**Figure 3:**
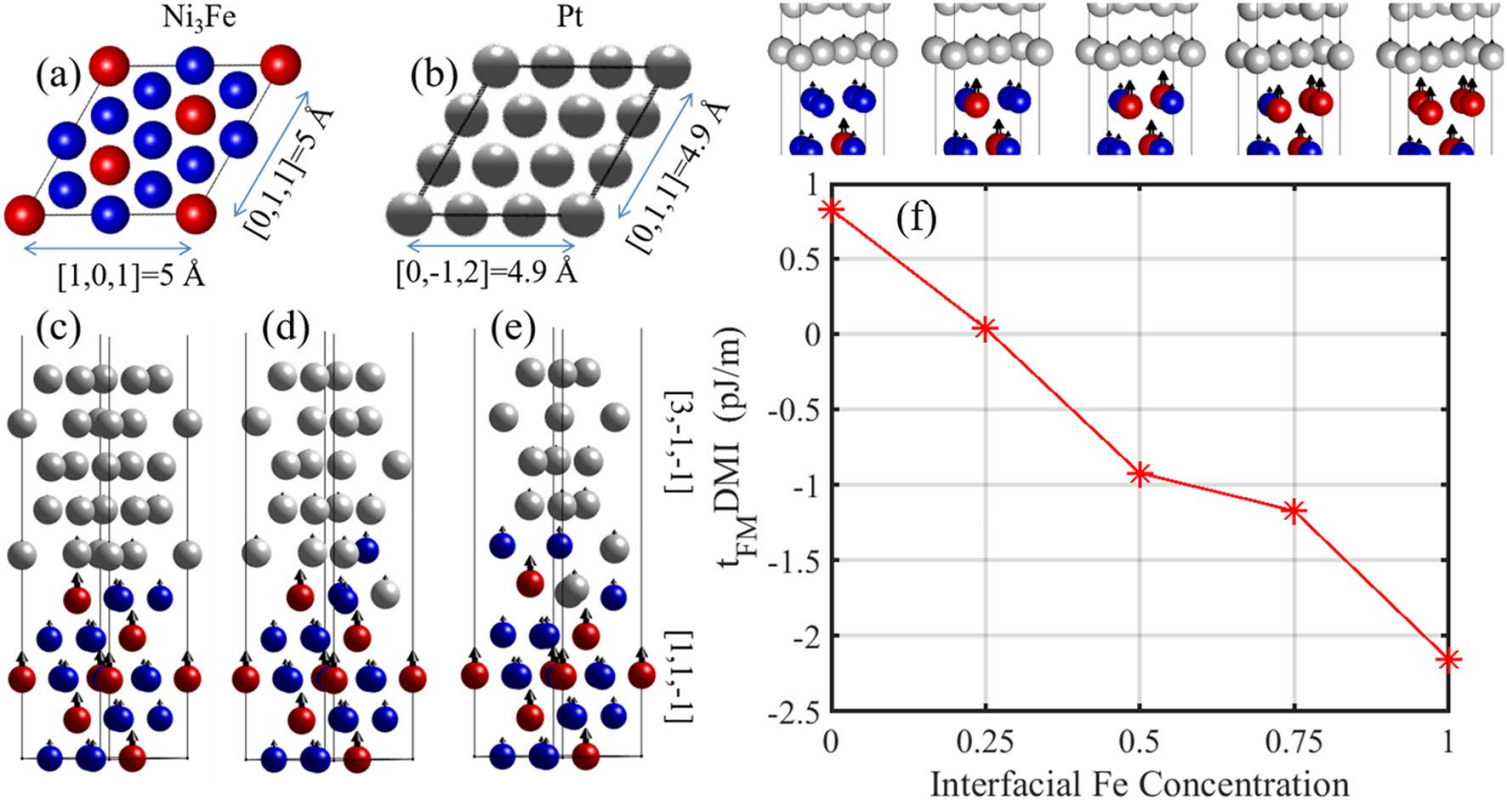
(**a**) Top view of L1_2_ Ni_3_Fe crystal structure with primitive lattice constant, 3.54 Å, where the blue (red) spheres denote the Ni (Fe) atoms. (**b**) Top view of FCC Pt crystal structure with primitive lattice constant, 2.81 Å. The in-plane lattice vectors are chosen so as to minimize the in-plane lattice mismatch between Ni_3_Fe and Pt to 2%. (**c**, **d**, **e**) Side views of the Ni_3_Fe(1 nm)/Pt(0.85 nm) bilayer device for various levels of substitutional disorders introduced at the interface via switching the Ni and Pt positions. (**f**) Calculated interfacial DMI versus concentration of Fe elements interfaced with Pt film.

The crystal structure of the Ni_3_Fe (1 nm) /Pt (0.85 nm) bilayer device and the calculated DMI values are presented in Fig. 3. In Fig. 3a and b we show the L1_2_ crystal structure of Ni_3_Fe and the FCC crystal structure of Pt grown along the $[\mathrm{1,1},\overline{1 }]$ and $[3,\overline{1 },\overline{1 }]$ directions, respectively. We build up supercells by aligning the in-plane [101] and [011] directions of Ni_3_Fe with the $[0,\overline{1 },2]$ and [011] directions of Pt as shown in Fig. 3a and b, respectively. This choice of growth direction results in a bilayer film with hexagonal crystal structure and a minimized lattice mismatch of 2% between the Ni_3_Fe and Pt films.

We have investigated the effect of two types of interfacial disorder on the iDMI. The first type corresponds to interdiffusion of Ni atoms in the Pt thin film, while the second corresponds to variation of the interfacial Fe concentration across the multilayer. In the first type of the disorder, shown in Fig. 3(c–e, we introduced substitutional interfacial disorder by switching the positions of Ni and Pt atoms at the interface. Figure 3c shows the ideal interface, while Fig. 3d and e illustrate lower and higher levels of disordered interfaces with one and two interfacial Ni atoms switched with the interfacial Pt, respectively.

We find that the iDMI for the ideal, one and two substitutional atoms is 0.05 pJ/m, 0.7 pJ/m and 2.5 pJ/m, respectively, indicating a monotonic increase of the DMI with interfacial disorder, which is opposite to the case of Pt/Co bilayer devices^13^. It should be noted that, since the interfacial disorder breaks the in-plane isotropy, we consider the average values of the DMI, i.e., DMI = (${D}_{xy}-{D}_{yx}$)/2.

The second type of interfacial disorder (Fig. 3f) in the [Ni_3_Fe/Pt] multilayer corresponds to substitution of interfacial Ni atoms with Fe atoms. Figure 3f displays the calculated DMI versus concentration of Fe atoms at the interface, which shows an almost linear decrease of DMI with the interfacial Fe concentration, resulting in a sign reversal of the DMI around 35% Fe concentration.

## Conclusion

In conclusion, we have studied the magnetic properties of sputter-deposited [Py(3 nm)/Pt(1 nm)]_×10_ symmetrical multilayer stacks. Thermal annealing at 300 °C and in inert atmosphere is used to induce interfacial Dzyaloshinskii–Moriya interaction in such symmetrical structures. Experiments based on Brillouin light scattering spectroscopy have been carried out to evidence the induction of the iDMI in the annealed samples. The estimated value of the induced iDMI in the annealed samples is approximately D = 0.47 mJ/m^2^. Structural characterizations showed that the annealing leads to a modified crystallinity and surface roughness, which can explain the underlying mechanism of the iDMI induction using this method. To further support our experimental observations, first principles calculations have been performed to show that increase of atomic interdiffusion at the interfaces of the magnetic/heavy metal layers enhances the iDMI from 0.05 to 2.5 pJ/m. The presented method can be used to tune the asymmetric exchange interactions in multilayers, which is an essential ingredient for spintronic and magnonic applications such as memory devices as well as nonreciprocal spin-wave elements.

## Methods

A multilayer stack with Ta(5 nm)/Pt(6 nm)/[Ni_80_Fe_20_(3 nm)/Pt(1 nm)]_×10_/Au(3 nm) structure was deposited on a thermally oxidized Si substrate at room temperature by DC magnetron sputtering. The base chamber pressure was 10^–8^ Torr, and samples were grown under an Ar pressure of 4 mTorr. The annealing of the sample has been carried out under Ar atmosphere (100 mTorr) at 300 °C for 1 h.

BLS measurements are performed using a *λ* = 532 nm laser beam by rotating the sample around a planar axis covering angles of incidence *θ* = 2.5°–75°, corresponding to the spin-wave (SW) wavenumber of *k*_sw_ = 1.03–22.8 rad/*μ*m given by *k*_sw_ = 4πsin (*θ*)/*λ* ^39^. The SWs frequency is determined by analyzing the backscattered light with a (3 + 3)-pass tandem Fabry-Pérot interferometer.

## Data Availability

The data that support the findings of this study are available from the corresponding authors upon reasonable request.
